# New Biomarkers for the Quick Detection of Acute Kidney Injury

**DOI:** 10.5402/2013/394582

**Published:** 2012-11-01

**Authors:** Abdulmuttalip Simsek, Volkan Tugcu, Ali Ihsan Tasci

**Affiliations:** Department of Urology, Bakırkoy Dr. Sadi Konuk Training and Research Hospital, Tevfik Saglam Street No. 11, Zuhuratbaba, 3400 Istanbul, Turkey

## Abstract

Acute kidney injury (AKI) is a common and strong problem in the diagnosis of which based on measurement of BUN and serum creatinine. These traditional methods are not sensitive and specific for the diagnosis of AKI. AKI is associated with increased morbidity and mortality in critically ill patients and a quick detection is impossible with BUN and serum creatinine. A number of serum and urinary proteins have been identified that may messenger AKI prior to a rise in BUN and serum creatinine. New biomarkers of AKI, including NGAL, KIM-1, cystatin-C, IL-18, and L-FABP, are more favourable tests than creatinine which have been identified and studied in several experimental and clinical training. This paper will discuss some of these new biomarkers and their potential as useful signs of AKI. We searched the literature using PubMed and MEDLINE with acute kidney injury, urine, and serum new biomarkers and the articles were selected only from publication types in English.

## 1. Introduction 

Acute kidney injury (AKI) remains a common and significant problem in the last decade [1]. Between 5% and 20% of critically ill patients in the intensive care unit (ICU) have an episode of AKI, with acute tubular necrosis (ATN) accounting for about 75% of cases [1–3]. Despite significant advances in both critical care and nephrology, the mortality rate of hospitalized patients with AKI has remained relatively unchanged at around 50% over the past few decades [4]. The most common causes of AKI are septic shock, ischemia, and nephrotoxins.

AKI has been defined conceptually as a rapid decline in glomerular filtration rate (GFR) that occurs over hours and days. It propels to a clinical syndrome characterized by a rapid decrease in renal excretory function, with the accumulation of products of nitrogen metabolism such as creatinine and urea clinically unmeasured waste products.

The described notions have led to a consensus definitions of AKI by the Acute Dialysis Quality Initiative. These RIFLE (risk, injury, failure, loss, end stage) criteria have been broadly supported with minor modifications by the acute kidney injury network (AKIN) [5, 6], and both definitions have now been validated in thousands of patients [7]. The AKIN group attempted to increase the sensitivity of the RIFLE criteria by recommending that a smaller change in serum creatinine (0.3 mg/dL) be used as a threshold to define the presence of AKI and identify patients with stage 1 AKI (RIFLE-Risk) [8]. In the AKIN classifications of AKI, a time of 48 h over which AKI occurs was proposed. However, there still remains much improvements to be made in increasing the sensitivity of current markers of AKI. Creatinine is currently the most widely used marker of renal function. Its use in the diagnosis of AKI remains a problem; however, as it often requires as much as a 50% loss in renal function before creatinine levels rise [5]. It is dependent on nonrenal factors independent of kidney function (age, sex, muscle mass, infection). Several medications (trimethoprim, cimetidine, and salicylates) alter the tubular secretion of creatinine, leading to changes in serum creatinine independent of GFR [9, 10].

These numerous problems with creatinine limit both clinical practice and the development of new therapeutics in AKI. Clinicians need tools that are not influenced by other clinical parameters or patient characteristics and that can identify losses in GFR soon after occurence. The ideal biomarker of AKI would be a substance that the kidney releases immediately in response to injury and that can be detected in the blood or urine without significant metabolism. This biomarker would be highly sensitive and specific for injury to the kidney. Extensive research efforts over this past decade have been directed at the discovery and validation of novel AKI biomarkers to detect injury prior to changes in kidney function and potentially to aid in the differential diagnosis of AKI.

The purpose of this paper is to review the relevant AKI biomarker literature ofwhich the search performed on MEDLINE/PubMed using the search terms “acute kidney injury, urine and serum new biomarkers” and articles were selected from all publication types in English.

## 2. Neutrophil Gelatinase-Associated Lipocalin (NGAL)

Human NGAL is a 25 kDa protein firstly identified bound to gelatinase in specific granules of the neutrophil. NGAL is a critical component of innate immunity to bacterial infection and is expressed by immune cells, hepatocytes, and renal tubular cells in various disease states [11, 12]. Its resistance to proteolysis further enhanced potential suitability as a clinical biomarker. It is synthesized and secreted by tubular epithelial cells of the proximal and distal segment. It is freely filtered by the glomerulus, undergoing rapid clearance by the proximal tubule via receptor binding and endocytosis. In healthy kidneys, it is barely detectable in either plasma or urine. However, in the setting of acute tubular injury, NGAL undergoes rapid and profound upregulation with large increases in both urine and plasma. Distinct from traditional markers of function such as creatinine, this rapid response enables NGAL to potentially identify injured kidney much earlier than was previously possible. The endogenous role of NGAL remains unclear. It seems to be involved with iron transportation to and from the proximal tubular epithelial cells, and animal studies demonstrate a renoprotective effect of exogenously administered NGAL in the setting of acute ischemic injury [13–15]. The appearance of NGAL in the urine preceded the appearance of the other urinary markers such as the tubular proteins N-acetyl-beta-D-glucosaminidase and beta_2_- migroglobulin. Studies in cultured human proximal tubule cells subjected to in vitro hypoxic injury confirmed the origin of NGAL from tubule cells. NGAL was also detected in the urine of mice in the early stage of cisplatin-induced nephrotoxicity [16]. These animal studies demonstrated that NGAL may represent an early, sensitive, and noninvasive urinary biomarker for ischemic and nephrotoxic kidney injury.

NGAL is the most extensively studied biomarker in AKI. Urinary and serum NGAL were demonstrated to be sensitive, specific, and highly predictive early biomarkers of AKI in children after cardiac surgery [17]. Seventy-one children undergoing cardiopulmonary bypass were studied and 20 children developed acute renal injury, but diagnosis with serum creatinine was only possible 1–3 days after cardiopulmonary bypass. In contrast, urine concentrations of NGAL rise from a mean of 1.6 microg/L at baseline to 147 microg/L 2 h after cardiopulmonary bypass, and the amount in serum increased from a mean of 3.2 microg/L at baseline to 61 microg/L 2 h after the procedure. NGAL demonstrated a near-perfect performance for identifying AKI after pediatric cardiac surgery with an area under the receiver operator characteristic curve (AUC_ ROC _) of 0.99 and 1.0 at 2 and 4 h after cardiopulmonary bypass (CPB), respectively [17]. Another study included forty children undergoing CPB. They were divided into group I, patients who suffered AKI grades II and III, and group II, patients who did not develop AKI. The results showed that there were highly significant differences in plasma NGAL levels between the groups; this significant difference started as early as 2 h after surgery, which reflects the potential role of plasma NGAL as an early biomarker in predicting AKI. The same results have been confirmed in many studies [18–20]. The another study of 374 children undergoing CPB, plasma, and urine NGAL significantly increased in AKI patients at 2 h after CPB and remained elevated for at least 48 hours, with the 2 h NGAL being the earliest and strongest independent predictor of AKI. 2-hour plasma and urine NGAL thresholds strongly correlated with length of hospital stay and severity and duration of AKI. [21]. The AUC for plasma and urine NGAL at various time points after CPB ranged from 0.88 to 0.97 indicating that both are excellent predictors of AKI. These decision have been confirmed in some studies of adults undergoing cardiac surgery [22–29]. However, results in adult cardiac surgery have been mixed and the AUC for the prediction of AKI have ranged widely from 0.61 to 0.96. The AUC_ ROC_ for early diagnosis of AKI by urinary NGAL has varied from 0.61 at 18 h after CPB [21] to 0.96 at 2 h after CPB [29]. Similarly, performance of plasma NGAL for the diagnosis of AKI has varied from an AUC_ ROC_ of 0.54 within 6 h of CPB [20] to 0.87 at 24 h after CPB [22]. The additional comorbidities in adult populations may be reflective of confounding variables such as age, preexisting kidney disease, bypass time, chronic illness, and diabetes [30].

NGAL may also represent early sensitive biomarker of AKI after contrast administrations for coronary angiography [31]. NGAL was measured in the serum and urine before and at 2, 4, 12, 24, and 48 h after contrast administration. They found a significant rise in serum NGAL 2 and 4 h after percutaneous coronary intervention (PCI), and a rise in urinary NGAL 4 and 12 h after PCI. A similar study of 25 patients with normal serum creatinine undergoing PCI due to unstable angina. There was a significant rise in serum NGAL after 2 and 4 hours. Urinary NGAL and urinary L-FABP followed the same pattern. Both markers increased significantly after 4 hours and remained elevated up to 48 hours after PCI [32]. Lastly, a study of 30 patients with normal serum creatinine undergoing coronary angiography were evaluated. There was a significant increase in serum NGAL level 4 hours and 24 hours after coronary interventions compared to the baseline value before coronary angiography [33].

NGAL is an early predictive biomarker of contrast-induced nephropathy (CIN) in children [34]. They studied 91 children with congenital heart disease undergoing elective cardiac catheterization and angiography with contrast administration. CIN, defined as a 50% increase in serum creatinine from baseline, was found in 11 subjects (12%), but detection using increase in serum creatinine was only possible 6–24 h after cardiac catheterization (CC). In contrast, significant elevation of NGAL concentrations in urine and plasma was noted within 2 h after CC. By multivariate analysis, the 2 h NGAL concentrations in the urine and plasma were found to be powerful independent predictors of CIN. With respect to other common nephrotoxins, early urinary NGAL measurements may be useful for the prediction of cisplatin, vancomycin, or cyclosporine-associated nephrotoxicity [35–37].

Urine and plasma NGAL levels also represent early biomarkers of AKI in an intensive care unite (ICU) [19, 38]. A multicenter study of serum NGAL was performed in 143 critically ill children with systemic inflammatory response syndrome (SIRS) or septic shock during the first 24 h of admission to the ICU. There was a significant difference in serum NGAL between healthy children, ill children with SIRS, and septic shock. The study was concluded that serum NGAL is a highly sensitive but nonspecific predictor of acute kidney injury in critically ill children with septic shock [38]. Several studies have examined NGAL levels in critically ill adult patients [39–47]. A 2010 study evaluated 88 ICU patient and found that an NGAL level of ≥150 nmol/L predicted AKI with 82% sensitivity and 97% specificity [44]. A recent study had 98 patients who were divided in two groups depending on the presence of sepsis. Fifty-six patients had sepsis, while forty-two patients were nonseptic. Among septic patients, subjects who developed AKI showed significant higher levels of NGAL [45]. A more study of 65 patients with septic shock were found that urine NGAL levels 12 hours before AKI diagnosis were a good predictor of AKI [48].

NGAL has been evaluated as a biomarker of delayed graft function (DGF) in a patient undergoing kidney transplantation. Hollmen and colleagues [49] demonstrate that donor urine NGAL is similarly useful to help with the preharvest prediction of DGF after renal transplantation from deceased donors. Specifically, they studied preharvest serum and urine NGAL levels in 99 consecutive, deceased donors, and followed the clinical courses of their 176 kidney recipients. They found that high preharvest urine NGAL levels were more common in those who developed prolonged DGF. In receiver operating characteristic (ROC) curve analysis, donor urine NGAL had poor utility for prediction of DGF (area under the curve 0.595) or prolonged DGF (area under the curve 0.616). However, increased donor urine NGAL was a significant risk factor for prolonged DGF. In others prospective studies, urine NGAL levels in sample collected on the day of transplant identified those who developed DGF with an AUC of 0.8-0.9 [50, 51].

NGAL is an early biomarker of AKI in children and adults in the following situations: postcardiopulmonary bypass, after contrast administration, nephropathy, in critically ill ICU patients, and delayed graft function in patients undergoing kidney transplantation.

## 3. Kidney Injury Molecule-1 (KIM-1)

Kidney injury molecule-1** (**KIM-1) is a putative epithelial cell adhesion molecule containing a novel immunoglobulin domain [52]. KIM-1 mRNA and protein are expressed at a low level in normal kidney but are increased dramatically in postischemic kidney [52–54]. KIM-1 has been identified as the first nonmyeloid phosphatidylserine receptor that confers a phagocytic phenotype on injured epithelial cells both in vivo and in vitro [55]. Urinary KIM-1 has been found to be an early indicator of AKI that compares favorably to a number of conventional biomarkers and tubular enzymes [53, 56].

KIM-1 is also a tissue and urinary biomarker for nephrotoxicant-induced kidney injury. Tissue and urinary expressions were measured with different nephrotoxic doses of cisplatin, folic aside, cadmium, gentamycin, mercury, and chromium [53, 57–60]. Marked increases in KIM-1 expression localized to proximal tubule cells were detected. As early as 1 day after cisplatin treatment, positive KIM-1 immunostaining, observed in the outer medulla of the kidney, and changes in urinary clusterin indicated the onset of proximal tubular injury in the absence of functional effects. Tissue KIM-1 was the most sensitive biomarker for detection of cisplatin-induced kidney damage [61].

KIM-1 is a biomarker of AKI in humans. Urine samples were collected from 32 patients with various acute and chronic kidney diseases, as well as from eight normal controls. There was extensive expression of KIM-1 in proximal tubule cells in kidney biopsies from six patients with biopsy confirmed acute tubular necrosis (ATN). Urinary KIM-1 levels were significantly higher in patients with ischemic ATN compared to levels in patients with other forms of acute renal failure or chronic renal disease. Adjusted for age, gender, and length of time delay between the initial insult and sampling of the urine, a one-unit increase in normalized KIM-1 was associated with a greater than 12-fold (OR 12.4, 95% CI 1.2 to 119) risk for the presence of ATN [56].

KIM-1 was also measured in 90 patients undergoing cardiac surgery. Thirty-six patients who developed AKI within 72 h after surgery. The AUCs for KIM-1 to predict AKI immediately and 3 h after operation were 0.68 and 0.65, 0.61 and 0.63 for NAG, and 0.59 and 0.65 for NGAL, respectively. Combining the three biomarkers enhanced the sensitivity of early detection of postoperative AKI compared with individual biomarkers: the AUCs for the three biomarkers combined were 0.75 and 0.78. This study demonstrated that combining multiple AKI biomarkers improved the overall predictive value [62]. A similar study described that preoperative KIM-1 and *α*-GST were able to predict the future development of AKI [63]. In hospital patients with AKI, urinary levels of KIM-1 that higher levels correlated with a higher odds ratio for dialysis requirement or hospital death [64]. This study demonstrated that urinary biomarker of AKI such as KIM-1 can predict adverse clinical outcomes in patients with AKI.

Renal KIM-1 expression is significantly increased in human kidney tissue among patients with a wide range of kidney diseases, including various types of glomerulonephritis, chronic allograft nephropathy, acute rejection, immunoglobulin A nephropathy, systemic lupus erythematosus, diabetic nephropathy, hypertension, and Wegener's granulomatosis [65]. Both renal and urinary KIM-1 correlate with kidney damage and negatively with renal function, but not with proteinuria. A recent study has explored urinary KIM-1 correlated with kidney function in kidney allograft recipients. Kidney transplant recipients showed significantly higher KIM-1 values than the healthy volunteers [66]. This study concluded that even a successful kidney transplantation is associated with kidney injury as reflected by elevated urinary KIM-1. In a similar study explored urinary biomarkers in 63 renal transplant recipients who require graft biopsy because of progressive worsening of kidney function. They reported that the rate of renal function decline significantly correlated with urinary KIM-1 expression after being followed for an average of 39.7 months. In kidney allograft recipients, urinary KIM-1 expression provides prognostic information in relation to the rate of renal function decline [67].

## 4. Cystatin C

Cystatin C is a protein produced by all nucleated cells. It is a polypeptide chain with 120 amino acid residues. It is freely filtered by the glomerulus, completely reabsorbed by the proximal tubules and is not secreted by the renal tubules [68]. In this way, some of the limitations of serum creatinine, effect of muscle mass, diet, gender, and tubular secretion may not be a problem with cystatin C and appear to rise one to two days earlier than serum creatinine [69]. Cystatin C is better marker of GFR than serum creatinine as demonstrated in several studies [70–77].

Serum cystatin C was measured in 25 children in the ICU. The ability of serum cystatin C to identify a creatinine rate a Schwartz creatinine clearance rate under 80 mL/min/1.73 m^2^ was better than creatinine (areas under the ROC curve: 0.85 and 0.79 for cystatin C and 0.63 and 0.62 for creatinine). This study concluded that serum cystatin C was better than serum creatinine to detect AKI in critically ill children [78]. Another study evaluated 85 patients at high risk to developing AKI. Forty-four patients developed AKI and the increase of cystatin C significantly preceded that of creatinine. Specifically, serum cystatin C increased already by more than 50% at 1.5 ± 0.6 days earlier compared to creatinine. Serum cystatin C demonstrated a high diagnostic value to detect AKI as indicated by area under the curve of the ROC analysis of 0.82 and 0.97 on the two days before the R-criteria (risk of kidney dysfunction) was fulfilled by creatinine [69].

Plasma and urine cystatin C were prospectively collected from 72 adults undergoing cardiac surgery. AKI was defined as a 25% or greater increase in plasma creatinine or renal replacement therapy within the first 72 hours following surgery. Plasma cystatin C and NGAL did not predict the development of AKI within the first 6 h following surgery. However, both urinary cystatin C and NGAL were increased in the 34 patients who later developed AKI. The urinary cystatin C at 6 h after ICU admission was the most useful for predicting AKI [23]. A recent study compared the sensitivity and rapidity of AKI detection by cystatin C level relative to creatinine level after 150 high-risk adult patients in the cardiac surgery. Serum creatinine level detected more cases of AKI than cystatin C level, 35% developed a ≥25% increase in serum creatinine level, whereas only 23% had a ≥25% increase in cystatin C level. This study concluded that cystatin C level was less sensitive for AKI detection than creatinine level [79]. Another study in 2011 examined presurgical values for cystatin C, creatinine, and creatinine-based estimated glomerular filtration rate (eGFR) in 1147 adults undergoing cardiac surgery for high risk AKI. Cystatin C also substantially improved AKI risk classification compared with creatinine. Presurgical cystatin C is better than creatinine or eGFR at prediction risk of AKI after cardiac surgery [80]. Abnormalities of thyroid function and glucocorticoid therapy may affecte cystatin C independently for kidney function. These situations are limitations to the use of cystatin C as a marker of GFR [81–83]. 

Early determination of allograft function and prognosis could lead to the development of therapies for kidneys with significant ischemia-reperfusion injury (IRI) and more effective recipient management, thereby improving outcomes. A study analyzed urine cystatin C in 91 patients who received deceased-donor kidney transplants to determine its peritransplant excretion pattern, utility for predicting delayed graft function, and association with 3-month graft function. Urine cystatin C/urine creatinine was highest in DGF for all time points. The area under the curve for predicting DGF at 6 h was 0.69 for urine cystatin C and 0.74 for urine cystatin C/urine creatinine. The urine cystatin C/urine creatinine ratio on the postoperative day was associated with 3-month graft function [84].

## 5. Interleukin-18 (IL-18)

IL-18 is a proinflammatory cytokine and the prototype of the chemokine superfamily. It is synthesized as an inactive 23 kDa precursor by several tissues including monocytes, macrophages, and proximal tubular epithelial cells. Animal studies explored the role of IL-18 in ischemic AKI [85–90]. Caspase-1 is a proinflammatory caspase via activation of the cytokine IL-18. Animal studies demonstrated that the caspase-1-mediated production of IL-18 plays a deleterious role in AKI [91].

DGF due to tubule cell injury frequently complicates deceased donor kidney transplants. Parikh showed that urinary NGAL and IL-18 represent early biomarkers for DGF. In patients with DGF, peak postoperative serum creatinine requiring dialysis typically occurred 2–4 days after transplant. Urine NGAL and IL-18 were elevated in the first day after transplant in patients with DGF. The receiver-operating characteristic curve for prediction of DGF based on urine NGAL or IL-18 at day 0 showed an area under the curve of 0.9 for both biomarkers [50]. The same author reported that increased levels of IL-18 in patients with AKI of varying etiology, especially those with delayed renal allograft function and ischemic ATN. In kidney transplant recipients, lower urinary IL-18 levels were associated with a steeper decline in serum creatinine concentrations postoperative days from 0 to 4 [92]. Immunohistochemical staining of protocol biopsies showed constitutive IL-18 expression in the epithelium of distal tubules with the induction of immunoreactivity in acute rejection patients where also proximal tubules, infiltrating leukocytes, and endothelium were strongly positive. Furthermore, serum levels of IL-18 were significantly elevated in patients with acute rejection of kidney allograft as compared to patients with uncomplicated outcome of kidney transplantation and subjects with acute tubulointerstitial nephropathy [93].

In a study of critically ill adult patients with acute respiratory distress syndrome, increased IL-18 was found to be an early marker of AKI and it was an independent predictor of death [94]. A subsequent study involved 451 patients, 86 of them developed AKI. The area under the receiver operating characteristic curve for urinary IL-18 predicting subsequent AKI within 24 hours was 0.62. It was found that urinary IL-18 remained independently predictive of composite outcome of death or acute dialysis within 28 days of ascertainment (odds ratio, 1.86). [95]. The predictive ability of urinary IL-18 has been demonstrated in one hundred thirty-seven critically ill children. The peak levels of IL-18 correlated with the severity of AKI. In nonseptic AKI patients, urinary IL-18 rises to a level higher than control levels 2 days prior to a significant rise in creatinine. Urinary IL-18 concentration from the first urine specimen was associated with AKI development within 48 h (odds ratio = 3.5) independent of the pediatric risk of mortality. This study concluded that urinary IL-18 rises prior to serum creatinine in nonseptic critically ill children, predicts severity of AKI, and is an independent predictor of mortality [96].

AKI is also common after cardiac surgery in adults and children. The study tested whether urinary IL-18 is a predictive biomarker for AKI in patients undergoing CPB. Serum creatinine was detected 48–72 h after CPB. In contrast, urine IL-18 increased at 4–6 h after CPB. These study results indicated that urinary IL-18 is an early, predictive biomarker of AKI after CPB [97]. AKI occurs commonly after pediatric cardiac surgery and associates with poor outcomes. 311 children undergoing surgery for congenital cardiac lesions to evaluate whether early postoperative measures of urine IL-18, urine NGAL, or plasma NGAL could identify which patient would develop AKI. 53 of them reached the primary outcome of severe AKI. Urine IL-18 and urine NGAL levels strongly associated with severe AKI. Elevated urine IL-18 and urine NGAL levels associated with longer hospital stay, longer intensive care unit stay, and duration of mechanical ventilation [98]. The same author also examined urine IL-18, urine, and plasma NGAL markers in adults cardiac surgery. They found that urine IL-18 and plasma NGAL at six hours were strongly associated with risk of AKI [99]. 

## 6. Liver-Type Fatty Acid Binding Protein (L-FABP)

L-FABP is a family of carrier proteins for fatty acids and other lipophilic substances such as eicosanoids and retinoids. FABP facilitates the transfer of fatty acids between extracellular and intracellular membranes. It was correlated with the degree of tubulointerstitial damage in a folic acid induced nephropathy [100]. FABP expression and urinary excretion have been described in several animal models of AKI [100–103]. In ischemic and cisplatin-induced AKI, L-FABP was increased in the urine before the increase in BUN. In cisplatin-induced AKI, urinary L-FABP levels increased exponentially even in the lowest dose group as early as 2 hours, whereas BUN levels increased at 48 hours. In ischemia reperfusion-induced AKI, BUN levels increased only in the 30-minute ischemia group 24 hours after reperfusion, however, urinary L-FABP levels increased more than 100-fold, even in the 5-minute ischemia group after 1 hour. In both AKI model, urinary L-FABP showed a better correlation with histology injury scores and GFR [103].

Urine L-FABP was measured in 40 pediatric patients prior to and following cardiopulmonary bypass surgery. Urinary L-FABP levels at 4 h after surgery were an independent risk indicator with the area under the receiver-operating characteristic curve 0.810, sensitivity 0.714, and specificity 0.684 for a 24-fold increase in urinary L-FABP. This study demonstrated that urinary L-FABP levels represent a sensitive and predictive early biomarker of AKI after cardiac surgery [104]. A recent study was undertaken to evaluate urinary L-FABP and NAG for AKI diagnosis in adult post-cardiac surgery patients. Twenty-eight patients developed AKI after surgery. Urinary L-FABP and NAG were significantly increased. However, ROC analysis revealed that the biomarkers' performance was statistically significant but limited for clinical translation, AUC-ROC for L-FABP at 4 hours 0.72 and NAG 0.75. Urinary L-FABP showed high sensitivity and NAG detected AKI with high specificity [105].

L-FABP was measured in 80 critically ill patients. Urinary L-FABP levels in patients with septic shock were significantly higher than severe sepsis without shock. Serum L-FABP levels did not have significant differences between patients with septic shock, severe sepsis, and healthy [106]. Another study was evaluated 145 septic shock patients complicated with established AKI. Urinary L-FABP measured at admission was significantly higher in the nonsurvivors of septic shock with established acute kidney injury than in the survivors and with an AUC for mortality prediction of 0.99 [107].

L-FABP was evaluated as a biomarker of renal ischemia in both human kidney transplant patients and a mouse model of AKI. In 12 living related kidney transplant patients after reperfusion of their transplanted organs, and intravital video analysis of peritubular capillary blood flow was performed. A significant direct correlation was found between urinary L-FABP level and peritubular capillary blood flow, ischemic time of the transplanted kidney as well as hospital stay [102]. A recent study examined the NGAL and L-FABP in diagnosis AKI in liver transplant recipients. Urinary NGAL was slightly elevated at 2 h in the non- AKI patients while rose and stayed high from 2 to 6 h in the AKI patients. However, urinary L-FABP rose transiently in both patients 2–120 h following surgery. The level of urinary NGAL presented differences at 2–6 h and urinary L-FABP at 4 h between AKI and non-AKI patients. This study concluded that urinary NGAL rather than L-FABP appeared to be a sensitive and specific marker of AKI in liver transplant recipients [108].

## 7. Conclusions 

There are multiple promising serum and urinary biomarkers, NGAL, KIM-1, cystatin C, IL-18, and L-FABP, which detect AKI before the rise in serum creatinine. However, serum creatinine is still the major determinant of kidney function. Determination of biomarkers of AKI in patients with AKI due to different causes, sepsis, ischemia, nephrotoxins, and contrast is important to monitoring. Establishing the optimal biomarkers for a clinical situation must require prospective validation in large numbers of patients with AKI and needs to be performed in different critically ill populations. These new biomarkers must be worked together to develop better definitions of AKI, probably in conjunction with serum creatinine, GFR, and urine output.
